# The ability of orthodontists and maxillofacial surgeons in predicting spontaneous eruption of mandibular third molar using panoramic serial radiographs

**DOI:** 10.1590/2177-6709.25.4.068-074.oar

**Published:** 2020

**Authors:** Mylena Ranieri Libdy, Nicole Melres Rabello, Leandro Silva Marques, David Normando

**Affiliations:** 1 Universidade Federal do Pará, Departamento de Ortodontia (Belém/PA, Brazil).; 2 Associação Brasileira de Odontologia, Departamento de Ortodontia (Belém/PA, Brazil).; 3 Universidade Federal dos Vales do Jequitinhonha e Mucuri, Departamento de Ortodontia (Diamantina/MG, Brazil).

**Keywords:** Third molar, Tooth extraction, Orthodontist, Oral and maxillofacial surgeon

## Abstract

**Objective:**

To evaluate the skill of orthodontists and oral/maxillofacial surgeons (OMFS) in providing a prognosis of mandibular third molars spontaneously erupted, through follow-up panoramic analysis.

**Methods:**

22 orthodontic patients treated without extraction, presenting spontaneously erupted mandibular third molars (n = 44) were analyzed through panoramic serial radiographs. The first panoramic radiograph was obtained just after orthodontic treatment (PR1), in patients aging from 13 to 19 years. A second panoramic radiograph (PR2), was obtained in average two years later. The radiographs were randomly analyzed by 54 specialists, 27 orthodontists and 27 OMFS, to obtain the opinion about the approach to be adopted to these teeth in PR1. Then, another opinion was collected by adding a serial radiograph (PR1+2).

**Results:**

The concordance of the answers was moderate for OMFS (Kappa 0.44; *p*< 0.0001) and significant for orthodontists (Kappa 0.39; *p*< 0.0001). In the analysis of the first radiograph (PR1) of the spontaneously erupted molars, OMFS indicated extraction in 44.5% of cases, while orthodontists indicated in 42%, with no difference between groups (*p*= 0.22). In PR1+2 analysis, orthodontists maintained the same level of extraction indication (45.6%, *p*= 0.08), while surgeons indicated more extractions (63.2%, *p*< 0.0001).

**Conclusions:**

Orthodontists and OMFS were not able to predict the eruption of the third molars that have erupted spontaneously. Both indicated extractions around half of the third molars. A follow-up analysis, including one more radiograph, did not improve the accuracy of prognosis among orthodontists and worsened for OMFS.

## RESUMO

**Objetivo:**

Avaliar a habilidade de ortodontistas e cirurgiões bucomaxilofaciais (CBMF) em propor um prognóstico para terceiros molares inferiores.

**Métodos:**

Foram analisados 22 pacientes tratados ortodonticamente sem extração, cujos terceiros molares inferiores irrompidos espontaneamente (n= 44) foram avaliados por meio de radiografias panorâmicas seriadas. A primeira radiografia foi obtida logo após o tratamento ortodôntico (RX1), entre 13 e 19 anos de idade. A segunda radiografia (RX2) foi avaliada dois anos depois, em média. As radiografias foram analisadas aleatoriamente por 54 especialistas, 27 ortodontistas e 27 CBMFs, para obter sua opinião sobre a abordagem a ser adotada na RX1. Em seguida, outra opinião foi coletada adicionando-se a segunda radiografia seriada (RX1+2).

**Resultados:**

A concordância das respostas foi moderada para os CBMFs (Kappa = 0,44; *p*< 0,0001) e significativa para os ortodontistas (Kappa = 0,39; *p*< 0,0001). Após analisar apenas a primeira radiografia (RX1) dos molares antes deles irromperem espontaneamente, os CBMFs indicaram extração em 44,5% dos casos; enquanto os ortodontistas, em 42%, sem diferença entre os grupos (*p*= 0,22). Na análise de RX1+2, os ortodontistas mantiveram o mesmo nível de indicação de extração (45,6%, *p*= 0,08), enquanto os cirurgiões passaram a indicar mais extrações (63,2%, *p*< 0,0001).

**Conclusões:**

Ortodontistas e CBMFs não foram capazes de predizer a erupção de terceiros molares por meio da análise de uma única radiografia panorâmica, indicando extrações em cerca da metade dos terceiros molares examinados. Uma análise de acompanhamento, incluindo mais uma radiografia, não melhorou a precisão do prognóstico entre os ortodontistas, e piorou entre os CBMFs.

## INTRODUCTION

Third molars are the most often impacted teeth,^1^ ^-^ ^4^ with a prevalence ranging from 9.5% to 39% among various populations^5^. Moreover, 75% of people receiving regular dental treatment have the third molars removed^6^. Lack of retromolar space,^7^ ^-^ ^10^ deficient mandibular growth^9^, distal eruption of dentition,^7^ condylar vertical growth direction,^9^ increased size of the crown,^7^ and late maturing^11^ have been reported as the most common causes of impaction.

The decision to preserve or remove third molars remains unclear to the clinician, partly because of the imprecision of prediction models on impacted molars reported in the literature.^12^ ^-^ ^17^ Thus, this decision seems to be centered on the preference of each speciality^18^, rather than a clinical approach based on scientific evidence. With so many controversies, prophylactic removal of third molars has been adopted under the assumption of preventing future damage,^19^ such as pericoronitis,^2^ osteitis, osteomyelitis,^20^ dentigerous cysts,^21^ caries in the distal of the second molar^22^, or root resorption in neighboring teeth.^23^ Furthermore, the tertiary crowding in adults^24^ ^-^ ^26^ and the risk of relapse after orthodontic treatment^26^ have been associated to the presence of third molars, although most studies have demonstrated that third molars have a negligible influence on the long-term changes occurring in the mandibular arch.^27^ ^,^ ^28^

On the other hand, some risks and complications^29^ may be associated with surgical removal of third molars, including alveolitis, injury to the inferior alveolar nerve,^30^ infections,^31^ and mandibular fracture.^32^ The most conservative approach is to carefully monitor asymptomatic third molars.^33^ This approach is based mainly in the absence of scientific evidence to justify prophylactic extraction.^17^ Monitoring should be performed every two years up to at least the age of 18.^8^

In order to examine the ability of experts on predicting the possibility of eruption of mandibular third molars (M3M), a study showed that orthodontists and oral/maxillofacial surgeons (OMFS) were unable to predict the prognosis of spontaneously erupted M3M after examining a single panoramic radiograph in 38.8% and 49.6% of the cases, respectively.^17^ The serial analysis of panoramic radiographs,^8^ a method widely used for clinical monitoring of orthodontic patients, might be able to increase the accuracy of this prediction. In this sense, the objective of this study is to evaluate the skills of orthodontists and OMFS in providing a prognosis for spontaneously erupted M3M by longitudinal monitoring through panoramic radiographs. 

## METHODS

This study was approved by the Human Research Ethics Committee of the Institute of Health Sciences of the Federal University of Pará (CEP-ICS / UFPA, protocol # 498024). Each dentist participating signed an Informed Consent Form. In addition, a Use of Database Agreement was signed by the orthodontist who provided patient clinical records and radiographs.

**Figure 1:**
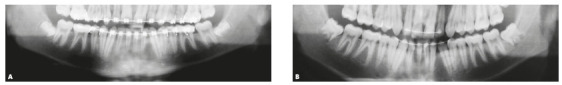
Panoramic radiograph after orthodontic treatment of the patient #6 at: A) 14 years and 9 months of age (PR1) and B) 16 years and 7 months (PR2). When examining the PR1, 64.7% of orthodontists and 29.4% of OMFS indicated the extraction of left M3M, while 64.7% of orthodontists and 35.3% of OMFS indicated the extraction of right M3M. By examining the two radiographs (PR1+2), 23.5% of orthodontists and 76.4% of OMFS indicated the extraction of the left M3M, while 17.6% of orthodontists and 70.6% of OMFS indicated extraction of the right M3M.

The sample included 22 patients, whose panoramic radiographs, two for each patient (n=44), were obtained from clinical records belonging to a single orthodontist in private practice. They were selected retrospectively from patients who had completed orthodontic treatment without extractions, and whose third molars had spontaneously erupted and were clinically asymptomatic. All patients had at least two panoramic radiographs: the first taken at the end of the orthodontic treatment (PR1, Fig 1A). A second image (PR2, Fig 1B) was obtained around two years after treatment, with the aim of monitoring the eruption of the mandibular third molars. Patients with agenesis, tooth loss, or extraction for orthodontic needs were previously excluded.

**Figure 2:**
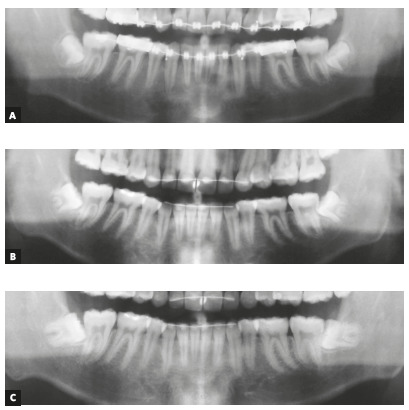
Panoramic radiograph at: A) the end of orthodontic treatment (PR1) and B) follow-up (PR2), of the patient used as a negative control case. Confirmation of the impaction was defined by a third radiograph (C), at 21.3 years.

Twelve men and 10 women, with a mean age of 14.5 years in the PR1 (13-16.6 years), and 16.8 years in the PR2 (15.5-19.6 years) were evaluated. A male patient, 15.4 years old in PR1 and 16.9 years in the PR2 (Figs 2A and 2B), whose third molars were severely impacted at 21.2 years (Fig 2C), was selected as a negative control. The inclusion of this case was carried out by a pilot study, in which five orthodontists unanimously indicated the impaction of the teeth on radiographs when the patient was 21.2 years.

Images of each radiograph was obtained using a digital camera (Canon EOS Digital Rebel EF-S 18-55; Canon Inc., Tokyo, Japan). The images were cropped in order to highlight the region of the mandibular third molars, ramus and angle of the mandible (Figs 1A and 1B). Radiographs were assembled randomly in a PowerPoint presentation (Microsoft, Redmond, USA). In addition, the age and sex of each patient were provided.

Fifty-four experts, 27 orthodontists and 27 OMFS, enrolled in the Regional Council of Dentistry of Pará (Brazil) were invited to provide a prognosis for the 44 mandibular third molars. The number of professionals enrolled in this study was based on a previous study^18^, which was shown to have enough power to detect intergroup differences. Three dentists declined to participate in the study (two orthodontists and one OMFS).

The experts first evaluated the panoramic radiographs at the end of orthodontic treatment (PR1) and were requested to indicate a prognosis for M3M bilaterally. The options included: monitoring, extraction, or other. Then, the experts examined the two radiographs together (PR1+2) at random, and indicated the prognosis again.

In cases where professionals adopted “extraction” as the preferred treatment for the tooth, they were asked to justify their decision with one of the following six options: 1) the presence or potential to develop a pathology; 2) second molar resorption risk; 3) it may lead to crowding; 4) caries risk; 5) tooth impacted or at risk of impaction; 6) other.

These analyses were performed for all 44 M3M that had spontaneously erupted and the negative control, totaling 46 M3M in 23 patients examined through 46 radiographs. Respondents were given sufficient time to respond to the questionnaire. 

To evaluate the method error, images of two patients (#13 and #15), including four M3M, were duplicated and were reassessed by each of the 54 examiners. The Kappa test was used to examine agreement of the intraexaminer and interexaminers responses. The intragroup and intergroups differences were evaluated by the chi-square test. Data were subjected to statistical analysis, with a significance level of 5%, using BioEstat 5.3 software (Mamirauá Institute, Belém/Pará, Brazil).

## RESULTS

The analysis of the cases duplicated, corresponding to four M3M, revealed a moderate agreement^34^ among the orthodontists (Kappa = 0.46) and OMFS (Kappa = 0.47) when the PR1 (Table 1) was evaluated. In the following analysis, in which a second radiograph was evaluated together with the first (PR1+2), the agreement increased significantly between orthodontists (Kappa = 0.65) and OMFS (Kappa = 0.67).

**Table 1:** Concordance of the answers related to the conduct adopted by oral/maxillofacial surgeons (OMFS) and orthodontists (ORTHO) compared to the third molars in the replicated cases (n=4) when examined the first panoramic radiograph (PR1) or two serial radiographs (PR1+2).

	PR1				PR1+2			
	ORTHO		OMFS		ORTHO		OMFS	
	RCC	Extraction	RCC	Extraction	RCC	Extraction	RCC	Extraction
RCC	72	10	53	11	62	11	37	9
Extraction	11	15	16	28	6	29	8	54
Kappa	0.46		0.47		0.65		0.67	
*p*-value	<0.0001		<0.0001		<0.0001		<0.0001	

RCC: radiographic clinical control.

In the analysis of the first panoramic radiograph (PR1), the OMFS indicated extraction in 44.5% of cases, while orthodontists indicated extraction in 42%, with no difference between them (*p*= 0.22, Table 2). In PR1+2, orthodontists maintained a similar level of extractions, when compared to the PR1 analysis (45.6%, *p*= 0.08), while the OMFS indicated more extractions (63.2%, *p*< 0.0001, Table 2). The Kappa agreement for only one radiograph, compared to using both radiographs (PR1+2), where the M3M erupted spontaneously (Table 2), was moderate for orthodontists (Kappa = 0.44) and considerable for OMFS (Kappa = 0.39). 

**Table 2:** Frequency agreement (Kappa), and difference (x^2^) of the responses indicated by orthodontists (ORTHO) and oral/maxillofacial surgeons (OMFS) on the clinical conduct adopted for the M3M that have spontaneously erupted (n = 44), when examined one (PR1) or two serial panoramic radiographs (PR1+2).

Prognosis	PR1		PR1+2		PR1 vs PR1+2			
	ORTHO	OMFS	ORTHO	OMFS	Concordance (Kappa)		PR1 vs PR1+2 (x^2^)	
	(n=27)	(n=27)	(n=27)	(n=27)	ORTHO	OMFS	ORTHO	OMFS
RCC	689 (58.0%)	657 (55.3%)	646 (54.4%)	436 (36.7%)	0.44 *p* < 0.0001	0.39 *p* < 0.0001	*p* = 0.08	*p* < 0.001
Extraction	499 (42.0%)	529 (44.5%)	542 (45.6%)	751 (63.2%)				
Others	0	2 (0.2%)	0	1 (0.1%)				
Total	1188	1188	1188	1188				
x^2^ ORTHO vs OMFS	1.63		74.54					
(*p*-value)	(*p*=0.22)		(*p*<0.0001)					

RCC: radiographic clinical control.

In examining the impacted M3M (Fig 2, Table 3), orthodontists indicated extraction in 79.6% of the responses when examining a single panoramic radiograph (PR1). For OMFS, extraction was pointed out on 74.1%, with no significant difference between the two groups of examiners (*p*= 0.81). When assessing PR1+2, 83.3% of orthodontists indicated extraction, while this option was indicated by 88.9% of the OMFS (*p*= 0.08). Compared to PR1, orthodontists and OMFS indicated, respectively, 3.7% (*p*= 0.8) and 14.8% (*p*= 0.08) more extraction when evaluating PR1+2 in cases of impaction. Kappa values ​​for the agreement between PR1 and PR1+2 was 0.52 for OMFS and only 0.38 for orthodontists (Table 3). 

**Table 3:** Frequency agreement (Kappa), difference (x^2^) of the responses indicated by orthodontists (ORTHO) and oral/maxillofacial surgeons (OMFS) on the clinical conduct adopted for the impacted M3M (n = 2) in the analysis of one (PR1) or two serial panoramic radiographs (PR1+2).

Prognosis	PR1 (n=2)		PR1+2 (n=2)		PR1 vs PR1+2			
	ORTHO (n= 27)	OMFS (n= 27)	ORTHO (n= 27)	OMFS (n= 27)	Concordance (Kappa)		PR1 vs PR1+2 (x^2^)	
					ORTHO	OMFS	ORTHO	OMFS
RCC	11 (20.4%)	14 (25.9%)	9 (16.7%)	6 (11.1%)	0.38 *p*=0.002	0.52 *p*<0.0001	*p* = 0.8	*p* = 0.08
Extraction	43 (79.6%)	40 (74.1%)	45 (83.3%)	48 (88.9%)				
Others	0	0	0	0				
Total	54	54	54	54				
X^2^ ORTHO x OMFS (*p*-value)	0.24 (*p*=0.81)				3.92 (*p*=0.08)			

RCC: radiographic clinical control.

In the PR1 analysis, the most prevalent justification for extraction among the orthodontists was *“risk of resorption of the second molar”* (45.5%), while for OMFS it was *“impacted tooth or at risk of impaction”* (38%). In PR1+2, both orthodontists and OMFS indicated *“impaction”* as their main justification (52.3% and 34.6%, respectively).

## DISCUSSION

The pathway of the third molars eruption have been the aim of several studies,^5^ ^,^ ^10^ ^,^ ^14^ but it has not yet been possible to develop a reliable predictive model.^12^ ^-^ ^17^ The prevalence of third molar impaction ranges from 9.5% to 39% among various populations.^5^ Third molars become more uprighted until 25 years of age, usually erupting between 18 and 24 years of age^6^. This fact is due to changes in the sagittal position, which has been found in posttreatment follow-up of orthodontic patients.

The present findings showed that when mandibular third molars erupt spontaneously, about 42% of orthodontists and 44.5% of OMFS indicated the extraction when evaluating a single panoramic radiograph taken at the end of orthodontic treatment (Table 2). However, when two serial radiographs from the same patient are examined, OMFS indicated significantly more extractions (63.2%, *p*< 0.0001), while orthodontists tended to maintain the same opinion. These data reveal that the longitudinal follow-up by analysis of serial panoramic radiographs did not improve the accuracy of prognosis among orthodontists, and worsened the prognosis for surgeons.

For one patient in which both M3M were clearly impacted in the long-term follow-up (Fig 2, Table 3), it was found that the majority of professionals (79.6% of orthodontists and 74.1% of OMFS) indicated the extraction of third molars after examining the first radiograph (PR1). By adding a second serial radiograph (PR1+2), orthodontists indicated the same amount of extractions (83.3%, *p*= 0.8), while OMFS indicated extraction in nearly 89% of cases, an increase of 14.8% compared to PR1, although not significantly different (*p*= 0.08). A larger sample size of impacted teeth could detect this tendency of change in the prognosis. However, this fact corroborates the results obtained in the analysis of cases in which third molars erupted spontaneously, where OMFS tended to indicate more extractions when two serial radiographs were evaluated (PR1+2), regardless of the final position of these teeth.

In summary, in cases of mandibular third molar impactions, the prediction ability of OMFS seems to improve slightly when a longitudinal series of two radiographs is presented. However, it is worsened when the third molars erupt spontaneously. Among the orthodontist, no difference was observed, and for cases of spontaneous eruption, a correct prognosis is similar to the probability of choice by chance (50%). Thus, it seems that OMFS indicate more surgical removal of third molars when analyzing radiographs in which these teeth are in a more advanced stage of development. 

Furthermore, since OMFS make decisions for more extractions than orthodontists in PR1+2, a lower inter-group agreement coefficient was found, when compared to the PR1 analysis.

When assessing the radiograph obtained at the end of the orthodontic treatment (PR1), the main reason among orthodontists to indicate extraction was the possibility of resorption of the second molar (45.5%) (Table 4). For OMFS, the main reason was the risk of impaction of third molars (38%). These findings may be associated with the pathway eruption of third molars with a mesial angulation.^35^ This angulation could lead to a more intimate contact with the adjacent tooth, leading professionals to plan a prophylactic extraction of M3M in order to prevent future pathological processes.^2^ ^,^ ^21^ ^,^ ^23^ Thus, despite the similar display of surgical removal between the orthodontists and OMFS, the reasons for the indication appear to be different.

**Table 4:** Reasons for choosing M3M extractions when orthodontists (ORTHO) and maxillofacial surgeons (OMFS) examined one (PR1) or two serial panoramic radiographs (PR1+2).

	PR1 (n=22)		PR1+2 (n=22)	
JUSTIFICATIONS	ORTHO (n=27)	OMFS (n= 27)	ORTHO (n=27)	OMFS (n= 27)
1. Resorption	312 (45.5%)	190 (23.0%)	187 (24.3%)	253 (20.3%)
2. Impaction	255 (37.1%)	314 (38.0%)	402 (52.3%)	431 (34.6%)
3. Tooth decay	37 (5.4%)	108 (13.0%)	79 (10.3%)	261 (20.9%)
4. Pathology	53 (7.7%)	111 (13.42%)	73 (9.5%)	208 (16.7%)
5. Crowding	27 (4.0%)	56 (6.8%)	28 (3.6%)	36 (3.0%)
6. Others	2 (0.3%)	48 (5.80%)	0	57 (4.5%)
Total	686	827	769	1246

With PR1+2 analysis, the reason reported by most respondents for the indication of extraction of M3M was the risk of impaction for both orthodontists (52.3%) and OMFS (34.6%, Table 4). It is likely that the advanced root development and the end of the retromolar space growth, widely reported factors of third molar impaction,^8^ ^,^ ^36^ have contributed to the reasons for their choice.

As the average age of the subjects in the present study was 14.5 years in PR1 and 16.8 years in PR2, a more conservative strategy would be to follow third molar development and position, by clinical and radiographic evaluation, until adulthood^2^. Also, active monitoring at 24-month intervals is recommended to allow the disclosure of clinical progression of periodontal disease^37^ and this was the time period evaluated in this study. In contrast, when these teeth are the cause of some painful symptoms, there is a general consensus for extraction.^38^

In asymptomatic cases, regular monitoring is required, making questionable the risks of maintaining the patient, taking into account the patient’s general state of health and the potential risk of systemic involvement.^39^ Whenever indicating extraction of third molars, dentists should have a justifiable reason, taking into account future treatment planning from an orthodontic, surgical, periodontal and/or prosthetic point of view.^40^ At the same time, a cost/benefit analysis should be carried out to justify the prophylactic removal of third molars.

The analysis of replicability of the cases studied showed greater concordance of responses when the two serial radiographs were examined (PR1+2) for both groups of evaluators (Table 1). This result seems to suggest that the higher the stage of development of the third molar, the greater agreement will be observed. However, this fact does not ensure a more accurate prognosis, whereas among OMFS, the level of error in the prognosis increased when the two radiographs were examined concurrently, at least for spontaneously erupted teeth. 

The evaluation of panoramic radiographs to suggest an accurate diagnosis was a limiting factor in this study. Although radiographs are currently used as the main instrument to observe and monitor third molars, this method does not replace clinical evaluation of the patient. Computed tomography (CT) is considered a more accurate technique to evaluate the involvement of anatomical structures, such as the mandibular canal, with the mandibular third molars. However, the ability of professionals to predict the eruption of these teeth using CT demonstrated that a three-dimensional image does not seem to change the prognosis established by specialists.^41^

The most important finding of this study is the information that clinical decision to extract M3M can be precipitated and often misguided when based on two-dimensional radiographic examinations. The results showed that even if the radiographs are taken longitudinally, the accuracy of prognosis is not increased. Furthermore, it seems to exist a need for prospective longitudinal studies evaluating the consequence of surgical removal of mandibular third molars, as well as for clinical and radiographic control.^38^

## CONCLUSIONS

These results allow us to conclude that orthodontists and oral/maxillofacial surgeons are not able to predict the prognosis of erupted mandibular third molars by examining a single panoramic radiograph. Both indicate extractions in almost half of spontaneously erupted teeth. Furthermore, the addition of a serial radiograph did not improve the accuracy of prognosis among orthodontists and worsened the accuracy for surgeons. Thus, it is suggested that these experts should re-evaluate their clinical protocol as well as radiographic guides used to establish a reliable prognosis on the eruption of third molars.
